# CK2 abrogates the inhibitory effects of PRH/HHEX on prostate cancer cell migration and invasion and acts through PRH to control cell proliferation

**DOI:** 10.1038/oncsis.2016.82

**Published:** 2017-01-30

**Authors:** Y H Siddiqui, R M Kershaw, E H Humphreys, E M Assis Junior, S Chaudhri, P-S Jayaraman, K Gaston

**Affiliations:** 1 School of Biochemistry, University Walk, University of Bristol, Bristol, UK; 2 Division of Immunity and Infection, School of Medicine, University of Birmingham, Birmingham, UK; 3 Queen Elizabeth Hospital, Birmingham, UK

## Abstract

PRH/HHEX (proline-rich homeodomain protein/haematopoietically expressed homeobox protein) is a transcription factor that controls cell proliferation, cell differentiation and cell migration. Our previous work has shown that in haematopoietic cells, Protein Kinase CK2-dependent phosphorylation of PRH results in the inhibition of PRH DNA-binding activity, increased cleavage of PRH by the proteasome and the misregulation of PRH target genes. Here we show that PRH and hyper-phosphorylated PRH are present in normal prostate epithelial cells, and that hyper-phosphorylated PRH levels are elevated in benign prostatic hyperplasia, prostatic adenocarcinoma, and prostate cancer cell lines. A reduction in PRH protein levels increases the motility of normal prostate epithelial cells and conversely, PRH over-expression inhibits prostate cancer cell migration and blocks the ability of these cells to invade an extracellular matrix. We show that CK2 over-expression blocks the repression of prostate cancer cell migration and invasion by PRH. In addition, we show that PRH knockdown in normal immortalised prostate cells results in an increase in the population of cells capable of colony formation in Matrigel, as well as increased cell invasion and decreased E-cadherin expression. Inhibition of CK2 reduces PRH phosphorylation and reduces prostate cell proliferation but the effects of CK2 inhibition on cell proliferation are abrogated in PRH knockdown cells. These data suggest that the increased phosphorylation of PRH in prostate cancer cells increases both cell proliferation and tumour cell migration/invasion.

## Introduction

The transcription factor PRH/HHEX (proline-rich homeodomain protein/haematopoietically expressed homeobox protein) is required during embryogenesis for the development of several organs including the heart, thyroid, pancreas and haematopoietic compartment (reviewed by Soufi and Jayaraman^1^). In the adult, PRH is expressed in multiple epithelial tissues and in haematopoietic cells. We have shown that PRH binds to specific DNA sequences near target genes including Vegfa and the VEGF receptor genes Vegfr-1 and Vegfr-2.^2^ Similarly, PRH directly regulates the CD105 gene encoding the TGFβ co-receptor protein Endoglin,^3^ and Goosecoid, a gene encoding a transcription factor that induces epithelial-mesenchymal transition in multiple cancer cell types.^4, 5^ PRH also regulates gene expression via protein–protein interactions with multiple transcription factors including c-Myc^6^ and SOX13.^7^ In addition, PRH regulates gene expression at the post-transcriptional level via an interaction with translation initiation factor eIF4E.^8^ Aberrant subcellular localisation of the PRH protein is associated with chronic myeloid leukaemia and some types of acute myeloid leukaemia, as well as with breast cancer and thyroid cancer.^8, 9, 10, 11^

Our previous work has shown that in chronic myeloid leukaemia cells PRH activity is controlled by Protein Kinase CK2 (Casein Kinase 2).^12, 13, 14^ CK2 is a ubiquitously expressed serine/threonine kinase important in the regulation of cell proliferation and cell stress responses.^15^ CK2 activity is increased markedly in benign prostatic hyperplasia (BPH) and prostatic adenocarcinoma.^16^ The CK2 tetramer comprises two regulatory β-subunits and two catalytic α-subunits. PRH interacts with the β-subunit of CK2 and is a target for phosphorylation by the α-subunit. Phosphorylation of PRH by CK2 results in the inactivation of PRH DNA-binding activity as well as proteasomal processing of hyper-phosphorylated PRH (pPRH) and the production of a pPRH fragment that inhibits the activity of full-length PRH.^12, 13^ Downregulation of PRH activity in chronic myeloid leukaemia cells by CK2 results in the de-repression of Vegfa and VEGF receptor genes and thereby promotes cell survival.^13^ CK2 phosphorylates two serine residues in PRH (S163 and S177)^12^ and the replacement of serine with cysteine at these positions in PRH S163C/S177C (PRH CC) prevents phosphorylation by CK2. Although wild-type PRH represses Vegfr-1 mRNA levels and CK2 over-expression counteracts this repression, CK2 over-expression is unable to counteract repression brought about by PRH CC.^13^ The replacement of these serines with glutamic acid in PRH S163E/S177E (PRH EE) produces a phosphomimic that fails to bind DNA or repress Vegfr-1 transcription.^13^

In prostate and breast epithelial cells, the regulation of Endoglin expression contributes to the control of cell motility by PRH.^3^ Moreover, over-expression of PRH in prostate cancer cells and breast cancer cells inhibits cell migration and inhibits the ability of prostate cancer cells to penetrate a layer of endothelial cells in *in vitro* extravasation experiments.^3^ Here we show that PRH is hyper-phosphorylated in BPH, prostatic adenocarcinoma and prostate cancer cell lines and that PRH phosphorylation in prostate cells is dependent on CK2 activity. PRH phosphorylation by CK2 inhibits prostate cancer cell migration and invasion. Moreover, PRH regulates the proliferation of prostate cells and the effects of CK2 inhibition on prostate cancer cell proliferation are mediated in large part at least by changes in PRH phosphorylation.

## Results

### PRH is phosphorylated by CK2 in prostate cells

**Figure 1:**
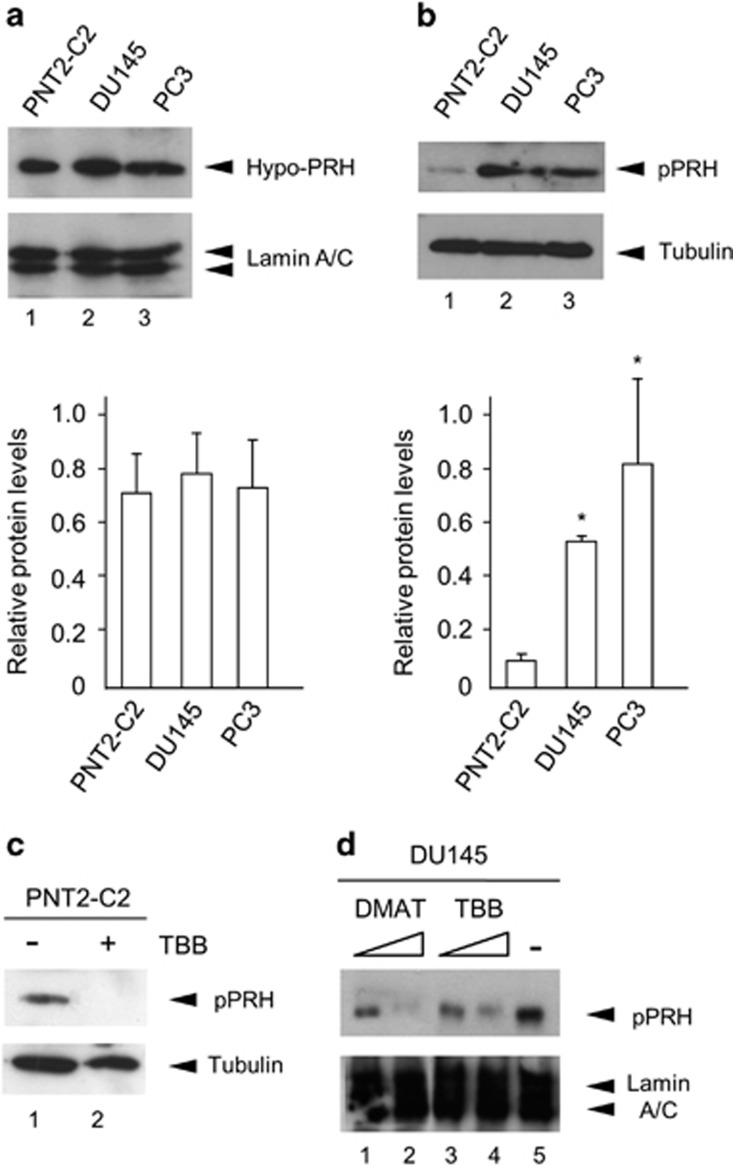
PRH is hyper-phosphorylated in two prostate cancer cell lines. (**a**, top) Representative western blot of hypophosphorylated PRH in normal immortalised PNT2-C2 prostate cells and two prostate cancer cell lines; DU145 and PC3. Hypo-PRH was stained with an anti-hypo-PRH mouse polyclonal antibody and Lamin A/C was used as a loading control. (bottom) Densitometric analysis of hypo-PRH levels in three independent experiments. (**b**, top) Representative western blot of hyper-phosphorylated PRH in the cell lines from **a**. pPRH was stained with an anti-pPRH rabbit polyclonal antibody and α-Tubulin was used as a loading control. (bottom) Densitometric analysis of pPRH levels in three independent experiments. DU145 *P=0.002, PC3 *P=0.015 (Student's unpaired *t-*test). (**c**) PNT2-C2 cells were treated with either 10% DMSO or 100 μm TBB in DMSO for 72 h. Western blotting was then used to examine the levels of pPRH (as above). Representative of three independent experiments. (**d**) DU145 cells were treated with (1) 50 μm DMAT, (2) 100 μm DMAT, (3) 50 μm TBB, (4) 100 μm TBB, in DMSO, or (5) 10% DMSO alone for 72 h. Western blotting was then used to examine the levels of pPRH (as above). Representative of three independent experiments.

We previously produced conformation-specific antibodies that recognise preferentially either hypophosphorylated PRH (hypo-PRH) or hyper-phosphorylated PRH (pPRH) and we used these antibodies to show that the inhibition of CK2 in leukaemic cells with specific inhibitors leads to loss of detection of pPRH.^13^ To examine the expression and phosphorylation status of PRH in prostate epithelial cells we made use of a normal immortalised prostate epithelial cell line (PNT2-C2 cells^17, 18^) and two well-characterised prostate cancer cell lines (DU145 and PC3 cells). Western blot analysis shows that hypo-PRH is present in all three cell lines (Figure 1a). PRH levels were quantified in several experiments by densitometry using Lamin A/C as a loading control (Figure 1a, lower panel). Hypo-PRH levels are equal across the three cell lines. In contrast, pPRH is weakly detected in PNT2-C2 cells and present at much higher levels in the prostate cancer cell lines (Figure 1b). Quantification relative to Tubulin as loading control confirms that pPRH is present at higher levels in the cancer cell lines (Figure 1b, lower panel). To confirm that CK2 is involved in the phosphorylation of PRH in prostate cells we treated PNT2-C2 cells for 72 h with the specific CK2 inhibitor TBB before western blot analysis using pPRH-specific antibodies. Phosphorylated PRH is present in the control cells but it is not detectable in the cells treated with TBB (Figure 1c). Tubulin antibodies confirm roughly equal protein loading. Similarly, treatment of DU145 cells with TBB or the CK2 inhibitor DMAT decreases PRH phosphorylation in a dose-dependent manner (Figure 1d). In these cells treatment with TBB also reduces phosphorylation of the unrelated CK2 target protein XRCC1 confirming that CK2 is inhibited (Supplementary Fig S1). In summary, these data are in agreement with our previous studies which showed that TBB and DMAT decrease PRH phosphorylation in other cell types and that purified CK2 can phosphorylate PRH *in vitro*.^12^ Moreover pPRH is present in the prostate cancer cell lines at higher levels than in normal immortalised prostate cells and CK2 activity is required for PRH phosphorylation in prostate cells.

### pPRH is elevated in BPH and prostatic adenocarcinoma

**Figure 2:**
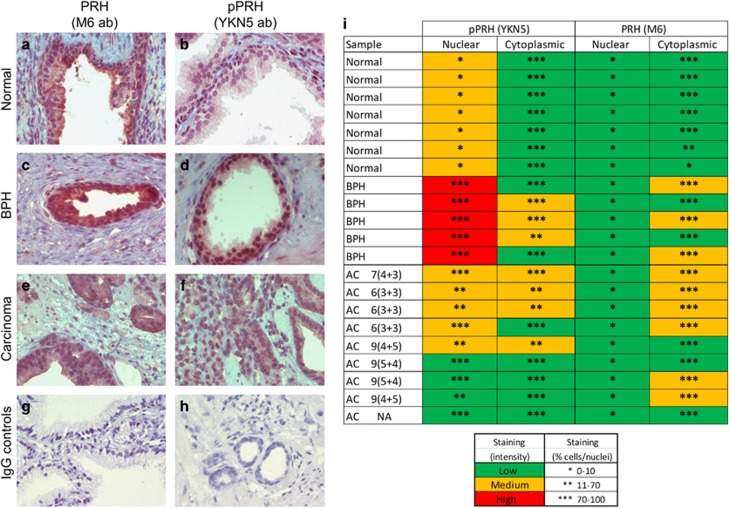
PRH and pPRH are present in normal prostate, BPH and prostate adenocarcinoma. Representative immunohistochemistry from normal prostate (**a**, **b**), BPH (**c** and **d**), and prostate adenocarcinoma (**e**, **f**) stained for total PRH using the M6 monoclonal antibody at 1:200 dilution (**a**, **c**, **e**) and pPRH using the YKN5 antibody at 1:750 dilution (**b**, **d**, **f**) as described in the text, and counterstained using Mayers haematoxylin. (**g**, **h**) Normal tissue stained using mouse and rabbit IgG, respectively (× 40 magnification). (**i**) A summary of the immunohistochemistry data for pPRH (YKN5) and PRH (M6) staining presented as the percentage of cells/nuclei that stain and the intensity of staining. AC, adenocarcinoma (with Gleason score); NA, not available.

To confirm that PRH is expressed in normal prostate tissue we performed immunohistochemistry on ethically obtained tissue sections from the University of Birmingham Biobank. In normal prostate, staining for total PRH reveals predominantly cytoplasmic PRH in the glandular epithelium with weak nuclear staining (Figure 2a). In contrast, staining for pPRH reveals weak cytoplasmic staining with little nuclear staining (Figure 2b). We conclude that PRH is expressed in normal prostate epithelial cells *in vivo* and that pPRH is also present in these cells. We next stained for PRH and pPRH in BPH and prostatic adenocarcinoma sections (Figures 2c and f). We scored PRH and pPRH cytoplasmic and nuclear staining respectively according to intensity and the number of positively staining cells in 14 BPH and prostatic adenocarcinoma sections and seven normal prostate sections. The data summarised in Figure 2i shows that nuclear pPRH staining is elevated in terms of intensity and the percentage of positively staining cells in 5/5 BPH. In contrast, in adenocarcinomas, the percentage of cells staining positively for nuclear pPRH is increased (9/9) but nuclear staining intensity is unchanged (5/9) or reduce (4/9). pPRH cytoplasmic staining appears to be similar in normal, BPH and adenocarcinomas, although there is a reduction in staining intensity in some BPH (3/5) and adenocarcinomas (4/9). Total PRH staining reveals that ~70–100% of cells show cytoplasmic PRH in normal prostate, BPH and adenocarcinoma. However, PRH cytoplasmic staining intensity is increased in BPH (3/5) and adenocarcinoma (6/9) compared with normal tissue (0/7). We conclude that nuclear pPRH levels are elevated in BPH and prostatic adenocarcinoma tissues and that total cytoplasmic PRH also appears to be elevated in these tissues.

### CK2 over-expression blocks the effects of PRH on cell migration

**Figure 3:**
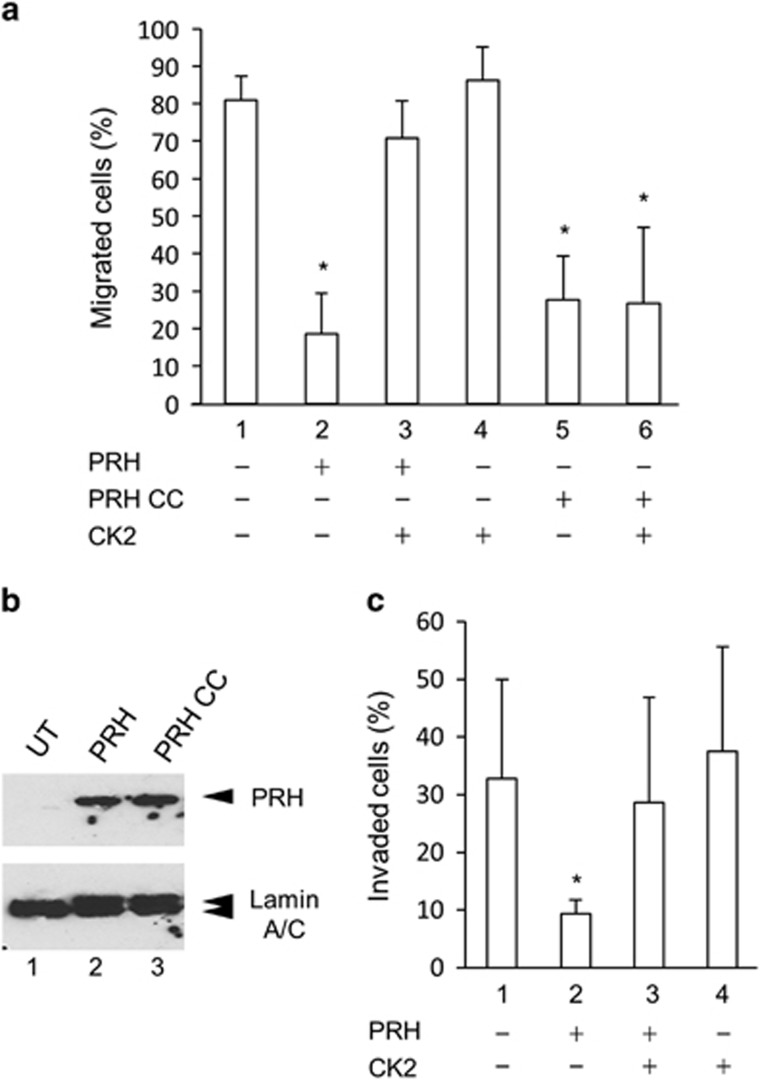
CK2 abrogates the effects of PRH on prostate cancer cell migration and invasion. (**a**) DU145 cells were transiently transfected with a GFP expression vector alone (1) or cotransfected with vectors expressing GFP, PRH or PRH CC and CK2 α- and β-subunits (2–6). Twenty four hours post transfection the cells were plated in Transwell chambers with a 2%:10% serum gradient. After 24 h the green cells on the top and bottom surfaces of the filter were counted using microscopy. Cells in 10 fields were counted in two independent experiments to determine the percentage of migrated cells. *P=0.04. (**b**) The expression of Myc-tagged PRH and PRH CC was examined using western blotting using an anti-Myc monoclonal antibody with Lamin A/C as loading control. (**c**) Invasion assays were performed as in **a** with a layer of Matrigel over the filter. *P=0.001.

We showed previously that PRH over-expression inhibits the migration of normal immortalised prostate PNT2-C2 cells and prostate cancer cells and significantly reduces the ability of DU145 cells to invade Matrigel.^3^ To determine whether the effects of PRH on cell motility are blocked by CK2 activity we made use of a non-phosphorylatable PRH mutant (PRH CC) that contains cysteine residues at the positions that are targeted by CK2 for phosphorylation (amino acids 163 and 177 in the PRH homeodomain).^13^ Over-expression of wild-type PRH in DU145 cells results in the inhibition of cell motility in a transwell cell migration assay (Figure 3a). However, co-expression of CK2α and CK2β blocks the inhibitory effects of PRH over-expression (Figure 3a). Over-expression of PRH CC also inhibits the migration of DU145 cells (Figure 3a). However, co-expression of CK2α and CK2β has no effect on the ability of PRH CC to inhibit cell migration (Figure 3a). Western blot analysis confirms that exogenous PRH and PRH CC are expressed at roughly equivalent levels in the transfected cells (Figure 3b). Over-expression of CK2α and CK2β alone has no effect of the migration of DU145 cells in this assay (Figure 3a) consistent with the hyperphosphorylation of PRH seen in these cells (Figure 1b). We repeated these experiments in PC3 cells and obtained identical results (Supplementary Fig S2) confirming that CK2 over-expression prevents PRH from inhibiting prostate cancer cell migration and that this requires the previously identified CK2 phosphorylation sites within the PRH homeodomain.

To determine whether CK2 over-expression also inhibits the effects of PRH on cell invasion we performed cell invasion assays in which Matrigel is layered over the transwell filter.^3^ As expected, DU145 cells are able to invade the Matrigel layer and migrate through the transwell filter (Figure 3c). However, while over-expression of PRH inhibits cell invasion, co-expression of PRH with CK2α and CK2β abrogates the inhibitory effects of PRH (Figure 3c). Over-expression of CK2α and CK2β alone has no effect on the invasive ability of DU145 cells in this assay (Figure 3c). Again this is consistent with the hyperphosphorylation of endogenous PRH seen in these cells (Figure 1b). We conclude that increasing CK2 levels also prevents exogenous PRH from inhibiting prostate cancer cell invasion.

### PRH knockdown induces cell migration and invasion and downregulates E-cadherin expression

**Figure 4:**
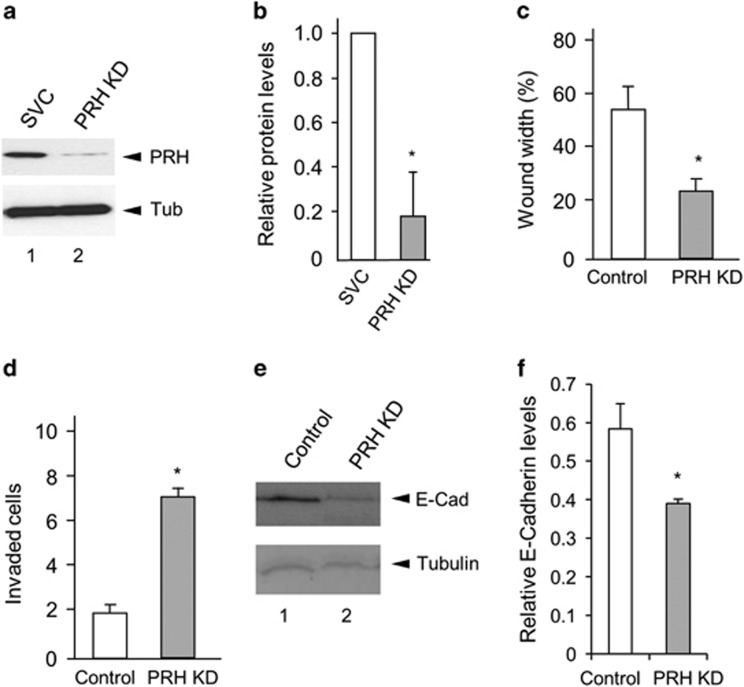
PRH knockdown induces invasion and downregulates E-cadherin protein levels. (**a**) PNT2-C2 cells were transfected with plasmids expressing either scrambled vector control shRNA or PRH shRNAs (PRH KD) and selected in media containing puromycin for 10 days. PRH expression was then examined by western blotting using an anti-PRH mouse monoclonal antibody with Tubulin as loading control. (**b**) Densitometric analysis of PRH protein levels at day 10 in puromycin selection in three independent experiments. *P=0.001. (**c**) PNT2-C2 control cells and PRH KD cells were plated to produce confluent monolayers that were then wounded with a pipette tip. After 24 h wound width was measured at multiple locations and plotted as percentage wound width remaining (*n*=3 independent control and KD cell lines, Student's *t-*test *P*=0.01). (**d**) Transwell invasion of PNT2-C2 cells through Matrigel over 48 h, (*n*=3 independent control and KD cell lines, Student's *t-*test *P*=0.004). (**e**) Western blot showing E-cadherin levels in PNT2-C2 cells and PNT2-C2 PRH KD cells. Representation of *n*=3. (**f**) Quantitative analysis of E-cadherin expression in three independent PRH KD cell lines (*n*=3, *Student's *t-*test *P*=0.03).

The data shown above suggest that PRH activity is compromised in prostate cancer cells due to increased phosphorylation. To investigate how loss of PRH activity alters the behaviour of normal prostate epithelial cells we knocked down PRH in PNT2-C2 cells using Prh shRNA (exactly as described previously^3^). PNT2-C2 cells were transfected with either a scrambled vector control shRNA plasmid or a combination of two PRH shRNA plasmids and selected for 10 days in puromycin (Figure 4a). Knockdown of PRH in PNT2-C2 cells resulted in increased cell migration in wound closure assays (Figure 4c). This is not the result of any effects of PRH knockdown on cell proliferation as the experiments were performed in the presence of hydroxyurea to inhibit cell division. Moreover, since over-expression of PRH in these cells resulted in decreased cell migration (Figure 3a), this increase in cell migration is unlikely to result from any off-target effects of the PRH shRNA. Surprisingly constitutive knockdown of PRH in PNT2-C2 cells also resulted in increased invasion (Figure 4d). This indicates that the PRH knockdown cells have a mesenchymal phenotype and suggests that the loss of PRH may alter cell-cell adhesion. In agreement with this hypothesis, the PNT2-C2 PRH KD cells express less E-cadherin protein compared with controls (Figures 4e and f).

### PRH regulates prostate cell proliferation

**Figure 5:**
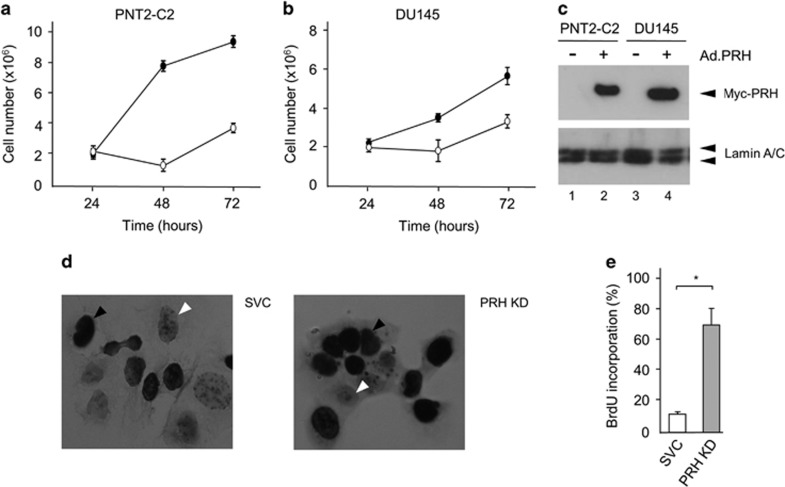
PRH inhibits the proliferation of normal immortalised prostate epithelial cells. (**a**) PNT2-C2 cells were infected with control adenovirus (filled circles) or an adenovirus expressing Myc-tagged PRH (open circles) at an MOI of 50. The number of viable cells was determined using an MTT assay at 24, 48 and 72 h post infection. Mean and s.d. *n*=3 independent experiments each performed in triplicate. (**b**) The experiment shown in **a** was repeated using DU145 cells. (**c**) Myc-PRH expression levels in the cells from **a**, **b** were determined as in Figure 3b. (**d**) BrdU staining of control and PRH KD cells (× 63 magnification). Representative BrdU-positive and -negative cells are indicated by filled and empty arrowheads, respectively. The cells were counterstained using haematoxylin. (**e**) The percentage of BrdU incorporation. Mean and s.d., *n*=3. *P=0.002.

To examine the effects of PRH on prostate cell proliferation, we made use of a previously characterised recombinant adenovirus expressing Myc-tagged PRH. PNT2-C2 and DU145 cells were infected with either a control adenovirus encoding β-galactosidase or the Myc-PRH adenoviral expression vector (Ad.PRH). The number of viable cells was then determined at increasing time points post infection using MTT assays. The MTT assay gives a quantitative analysis of cell number and is therefore superior to qualitative cell viability assays. In both cell lines, PRH over-expression results in a significant reduction in the number of viable cells (Figures 5a and b). Western blot analysis confirms the expression of Myc-tagged PRH (Figure 5c). To confirm that PRH regulates prostate cell proliferation we made use of the PNT2-C2 PRH KD cells described above. The proliferation of PNT2-C2 PRH KD cells and control cells was assayed using bromodeoxyuridine (BrdU) incorporation assays. Control cells and PRH knockdown cells were incubated with BrdU for 15 h and the cells were then fixed and stained with an anti-BrdU antibody. The percentage of BrdU-positive cells increases from ~15% in the control population to ~75% in PRH knockdown population (Figure 5d,e). These results demonstrate that PRH over-expression inhibits prostate cell proliferation, while PRH knockdown increases the proliferation of PNT2-C2 cells. Knockdown of PRH in DU145 was not performed since these cells are effectively PRH-depleted due to increased PRH phosphorylation and knockdown is therefore unlikely to increase their proliferation.

**Figure 6:**
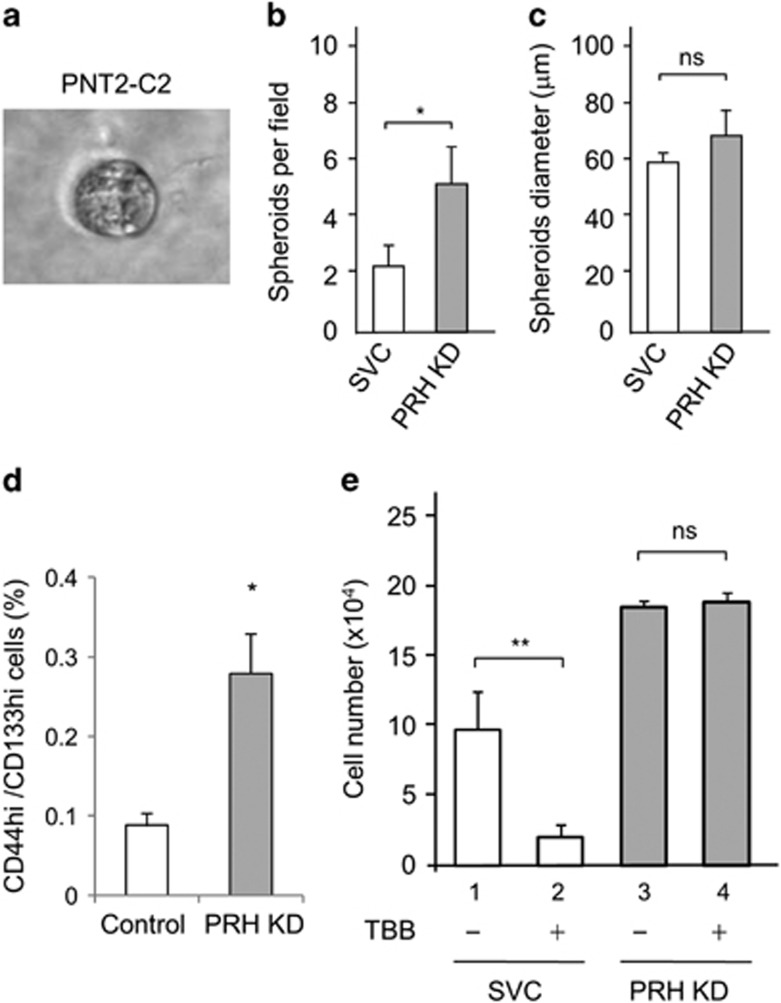
PRH knockdown increases the number of colony forming cells. PNT2-C2 cells and PNT2-C2 PRH KD cells were mixed with Matrigel 1:1 v/v (on ice) and plated in 0.5 ml aliquots in a 24-well plate. After 15 days in culture phase-contrast images were taken using a Leica DMPXA/DMRBE microscope with Volocity 5.5.1. A representative PNT2-C2 spheroid is shown (**a**) at × 100 magnification. The number of spheroids in 10 fields (**b**) and their diameter (**c**) was determined using ImageJ. Mean and s.d. are shown in each case (*n*=3 independent knockdowns). *P=0.04 and ns—not significant. (**d**) The percentage increase in the CD44hi/CD133hi population determined using flow cytometry. Means and s.d. for *n*=3. *P=0.01. (**e**) Control scrambled vector control shRNA plasmid cells (empty bars) and PRH knockdown cells (filled bars) were treated with 10% DMSO or 100 μm TBB in DMS0 for 72 h. The number of viable cells was then determined using MTT assays. Mean and s.d., *n*=3. *P=0.01 and ns, not significant.

To assess the role of PRH in an assay with more relevance to the native tissue environment compared with a monolayer culture, we next examined the effects of PRH knockdown on the formation of spheroids in a colony formation assay. PNT2-C2 cells suspended in Matrigel form spheroidal structures^17^ reminiscent of glandular epithelium (Figure 6a). We plated equal numbers of PNT2-C2 cells and PNT2-C2 PRH knockdown cells in Matrigel and counted the number of spheroids formed after 14 days in culture. PRH knockdown resulted in a significant increase in the number of spheroids formed (Figure 6b). Since each colony arises from a single cell this indicates that the reduction in PRH levels results in an increase in the number of colony forming cells present in the PNT2-C2 population rather than from a change in cell proliferation. Interestingly, the diameter of the spheroids produced by the PRH knockdown cells is unaltered (Figure 6c). Thus PRH knockdown has little or no effect on spheroid growth from the initial colony forming cell. In support of these conclusions flow cytometry showed a significant increase in the number of CD44+/CD133+ cells indicative of the self-renewing stem cell population able to form colonies (Figure 6d). Thus robust knockdown of PRH in these cells results in increased cell proliferation and an increase in the number of cells capable of colony formation, as well as increased invasion.

### PRH mediates the effects of TBB on the proliferation of PNT2-C2 cells

CK2 activity is often elevated in cancer cells and CK2 inhibition has been proposed as a novel treatment for prostate cancer.^19^ To determine whether the inhibition of CK2 activity alters the proliferation of PNT2-C2 cells and to investigate whether any effects of CK2 inhibition are dependent on changes in PRH phosphorylation, we treated control and PNT2-C2 PRH knockdown cells with the CK2 inhibitor TBB. After 72 h, we determined the number of viable cells using an MTT assay. Treatment of control PNT2-C2 cells with TBB resulted in a significant reduction in the number of viable cells (Figures 6e, 1 and 2). This was accompanied by the loss of pPRH (Figure 1c). As expected, untreated PNT2-C2 PRH knockdown cells proliferated more than untreated PNT2-C2 cells over 72 h resulting in an increase in the number of viable cells (Figures 6e, 1 and 3). However, treatment of the knockdown cells with TBB had no effect on the number of viable cells (Figures 6e, 3 and 4). These data show that TBB treatment inhibits the proliferation of PNT2-C2 cells and they suggest that changes in PRH phosphorylation mediate the effects of TBB on these cells. It is not possible to examine the effects of TBB on pPRH levels in the PRH knockdown cells due to the low level of pPRH in these cells.

## Discussion

Aberrant intracellular localisation of PRH and/or altered PRH levels have been observed in a number of disease states including breast and thyroid cancer.^10, 11^ This suggests that loss or reduction of PRH activity is associated with the increased proliferation of breast and thyroid cancer cells. Our previous work showed that phosphorylation of PRH by CK2 abolishes the DNA-binding activity of PRH.^12^ We showed that this prevents PRH from regulating transcription and abrogates the effects of PRH on the proliferation of leukaemic cells.^13^ Here we have shown that nuclear pPRH is increased in BPH and prostatic adenocarcinoma compared with normal tissue and that pPRH levels are increased in prostate cancer cell lines compared with normal immortalised prostate epithelial cells. This suggests that in BPH and prostate cancer cells PRH activity is inhibited by increased phosphorylation. Since PRH inhibits the proliferation of prostate cells, this is in keeping with the increased proliferation of BPH and prostate cancer cells. The increase in pPRH is also in keeping with the reported increase in CK2 activity in BPH and prostate cancer cells.^16^ Total PRH staining is more intense in the cytoplasm in the BPH and adenocarcinoma samples. This suggests that the cytoplasmic/nuclear distribution of PRH is also altered in BPH and prostate adenocarcinoma and this could also prevent PRH from regulating transcription.

Our recent work has shown that decreasing PRH levels in normal prostate and breast epithelial cells can induce a migratory phenotype.^3^ Moreover, PRH over-expression can inhibit the migration of prostate and breast cancer cells, inhibit the invasion of an extracellular matrix by these cells and inhibit the passage of prostate cancer cells through a layer of normal human endothelial cells in an *in vitro* extravasation assay.^3^ In leukaemic cells, PRH is known to interact with the regulatory β-subunit of CK2 via a protein–protein interaction and phosphorylation of PRH by CK2 results in PRH inactivation.^12^ Here we have shown that the inhibition of CK2 in normal immortalised prostate epithelial PNT2-C2 cells results in the loss of detection of pPRH. This suggests that CK2 phosphorylates PRH in these cells. Over-expression of CK2 in prostate cancer cells prevents PRH from inhibiting cell migration and cell invasion but has no effect on the ability of a non-phosphorylatable PRH mutant to inhibit cell migration. In marked contrast, knockdown of PRH in non-invasive PNT2-C2 cells induces cell migration and cell invasion and this is accompanied by a decrease in E-cadherin protein levels. These data are consistent with the observation of increased levels of pPRH in migratory and invasive prostate cancer lines and in prostatic adenocarcinoma. Taken together these observations suggest that PRH controls the migratory behaviour of prostate cells and that the increased PRH phosphorylation seen in the prostate cancer cells contributes to their increased migration and invasion.

PRH is known to regulate cell proliferation in multiple cell types.^1^ PRH over-expression inhibits the proliferation of CML cells and we have shown that over-expression of CK2 abrogates the negative effects of PRH over-expression on these cells.^2^ Here we have shown that PRH over-expression inhibits the proliferation of normal immortalised prostate cells and prostate cancer cells. Moreover, PRH knockdown increases the proliferation of normal immortalised prostate cells. This suggests that PRH expression and PRH phosphorylation are important in the control of prostate cell proliferation as well as prostate cell migration. In normal immortalised prostate cells knockdown of PRH increases the number of cells that are capable of forming spheroids in Matrigel and the number of cells displaying stem cell marker proteins (the CD44hi/CD133hi population^20, 21^). This suggests that the downregulation of PRH increases the number of stem cells in the population capable of colony formation. The resulting spheroids are identical in size to the spheroids formed by control cells. Thus the controls that regulate spheroid formation are not disrupted by the loss of PRH. Recent evidence shows that spheroid formation and prostate cancer stem cells are linked to E-cadherin expression and epithelial-mesenchymal transition.^22, 23^ Since knockdown of PRH in normal PNT2-C2 cells also results in decreased E-cadherin expression, as well as increased spheroid formation, we conclude that PRH normally regulates self-renewal, cell proliferation and cell-cell contacts in these cells. Moreover, phosphorylation of PRH in prostate tumour cells is likely to contribute to their increased invasive potential and their increased ability to form cancer stem cells.

In conclusion, we propose that in both normal and transformed prostate epithelial cells PRH expression limits cell proliferation and cell migration and invasion. Although treatment of normal prostate cells with the specific CK2 inhibitor TBB results in a decrease in cell proliferation, TBB has little or no effect on the proliferation of PRH knockdown cells. This suggests that the inhibitory effects of CK2 inhibitors on the proliferation of these cells are mediated by PRH. This is important since CK2 activity is elevated in prostate cancer and other disease states and the inhibition of CK2 is a potential treatment for these diseases.

## Materials and methods

### Cell culture

PNT2-C2 cells were a kind gift from Professor Norman J Maitland (University of York). PC3 and DU145 were obtained from the ATCC (Manassas, VA, USA). Cells were cultured in RPMI-1640 supplemented with 10% fetal bovine serum, 2mm l-glutamine and 1% penicillin/streptomycin and maintained in a humidified atmosphere at 37 °C and 5% CO_2_. In some experiments cells were also incubated with TBB (4,5,6,7-tetrabromo-2-azabenzimidazole) or DMAT (2-Dimethylamino-4,5,6,7-tetrabromo-1H-benzimidazole) (Sigma, St Louis, MO, USA). Three dimensional culture of PNT2-C2 cells was performed as described by Maitland.^17^ Cells were suspended in growth media (6 × 10^4^ cells/ml) then mixed with Matrigel (Sigma) on ice in a 1:1 (v/v) ratio and plated into 24-well plates. The Matrigel was set by incubating at 37 °C for 30 min, after which growth media was added to each well and changed every 3 days. Images were obtained using a phase-contrast Leica (Wetzlar, Germany) DMIRB inverted microscope at × 10 magnification, using the Image Acquisition software Volocity 5.5.1 (Perkin Elmer, Akron, OH, USA). The diameter of the spheroids was measured using ImageJ software (NIH, Bethesda, MD, USA).

### Transient transfection and expression plasmids

Cells were transiently transfected using TransIT (Mirus, Madison, WI, USA) according to the manufacturer's protocol. The PRH expression vectors pMUG1-Myc-PRH, pMUG1-Myc-PRH N187A, pMUG1-Myc-PRH F32E, pMUG1-Myc-PRH S163C/S177C and pMUG1-Myc-PRH S163E/S177E have been described.^13, 24, 25^ The CK2 expression vectors pRc/CMV-CK2α-HA and pRc/CMV-CK2β-HA were a kind gift from Professor D Litchfield (University of Western Ontario). Plasmids encoding short hairpin RNAs (shRNAs) shRNAPRH49, shRNAPRH51, and control shRNA were supplied by Origene (Rockville, MD, USA).

### Immunohistochemistry

Immunohistochemistry was performed on formalin-fixed paraffin-embedded human prostate tissues (normal, hyperplasia, low- or high-grade carcinoma). Tissues were ethically obtained from the Queen Elizabeth Hospital Birmingham pathology archive through the Human Biobank Repository Centre. The tissues were stained with the Vector ImmPress Excel anti-mouse Ig or anti-rabbit Ig peroxidase kits (MP-7602 or MP-7601, respectively, Vector Laboratories (Burlingame, CA, USA) and visualised with the ImmPact NovaRed peroxidase substrate (Vector Laboratories) according to the manufacturer's instructions. Staining was performed as follows: tissues were dewaxed in xylene (PFM Medical, Cologne, Germany) and then rehydrated in graded alcohols (VWR) and then distilled water. Antigen retrieval was performed with high pH buffer (Vector Laboratories) and the sections were incubated in Bloxall solution (Vector Laboratories) for 10 min at 20 °C to block endogenous peroxidase activity and then in 2.5% horse serum (Vector Laboratories) for 20 min at 20 °C. The primary antibodies (mouse anti-human PRH (M6) 1:200 or rabbit anti-human PRH (YKN5) 1:750) diluted in 2.5% horse serum were added to the sections and incubated overnight at 4 °C. The sections were then washed in Tris-buffered saline pH 7.2 (Pierce, Paisley, UK) for 5 min and incubated with amplifier antibody for 15 min at 20 °C. After washing in Tris-buffered saline pH 7.2 for 5 min the sections were incubated with the ImmPress Excel tertiary antibody for 30 min at 20 °C. The sections were washed in Tris-buffered saline pH 7.2 for 5 min and the NovaRed peroxidase substrate was added for 5 min. The reaction was stopped by washing in distilled water for 5 min and the sections counterstained using Mayers haematoxylin (PFM Medical) for 30 s. The sections were mounted using DPX (Cell Path, Mochdre, UK) and left to dry before being visualised on a Zeiss (Oberkochen, Germany) microscope. Images were acquired using AxioVision v4.3 software (Zeiss).

### Western blot analysis

Whole cell extracts were prepared in TES buffer (1% SDS, 2 mm EDTA, 20 mm Tris-HCl pH 7.4) as described previously.^3^ Antibodies that recognise total PRH, hyper-phosphorylated PRH and hypophosphorylated PRH have been described previously.^13^ Lamin A/C (H-110) and Tubulin (H-235) antibodies were from Santa-Cruz (Dallas, TX, USA). The antibody for phosphoXRCC1 was a kind gift from Dr Grant Stewart (University of Birmingham). Densitometric analysis was performed using Quantity One 4.6 software (BioRad, Hercules, CA, USA).

### Cell migration and invasion assays

Chemotaxis assays were performed by seeding cells onto 8 μm Boyden chambers (Greiner Bio-One, Frickenhausen, Germany) in RPMI-1640 with 2% fetal bovine serum. The chambers were placed into 24-well plates containing RPMI-1640 with 10% fetal bovine serum to create a serum gradient. After incubation in a humidified atmosphere at 37 °C and 5% CO_2_ for 24 or 48 h the cells were fixed with 4% paraformaldehyde (Fisher) and stained with 2 μg/ml bisbenzimide (Sigma). Cells on the top and bottom of the membrane were counted using a Leica Q550 inverted epifluorescence microscope or Zeiss Axioplan 2. Invasion assays were performed as above except that Matrigel (BD Biosciences) was added to the chambers and left at 37 °C for 1 h to solidify before seeding. For wound closure assays a confluent layer of cells was wounded using a pipette tip before fresh culture media containing 2 μm hydroxyurea (Sigma) was added to block cell proliferation. After 24 h phase-contrast images were obtained at × 100 magnification, using Axiovision with an inverted bright-field microscope and the migrated distance was measured at 20 points along the wound edge using ImageJ.

### MTT assays

MTT (3-(4,5-dimethyl-2-thiazolyl)-2,5-diphenyl-2H-tetrazolium bromide) cell viability assays were performed as described previously.^13^ Quantification was carried out on a VERSAmax microplate reader (Molecular Devices, Sunnyvale, CA, USA) at 540 nm using SOFTmax PRO 3.1.2 (Molecular Devices, San Diego, CA, USA). In PRH over-expression experiments cells were treated with either empty adenovirus or an adenovirus expressing Myc-tagged PRH ^26^ at a multiplicity of infection (MOI) of 50 and incubated in a humidified atmosphere at 37 °C and 5% CO_2_ for up to 72 h.

### BrdU incorporation assays

Cells growing on cover slips were incubated in media containing 10 μm BrdU (Sigma) for 15 h. After fixation in 4% paraformaldehyde (10 min at 20 °C) the cells were incubated with hydrogen peroxide for 5 min at 4 °C. The cells were then incubated with 62 500 Units Benzonase (Sigma) and 1 mm MgCl_2_ at 37 °C for 2 h with shaking. The cells were then incubated with an anti-BrdU antibody (1:500, Sigma B8434) in 1% bovine serum albumin/phosphate-buffered saline overnight at 4 °C followed by incubation with horse anti-mouse biotin conjugate (1:200, Vector Laboratories) in 1% bovine serum albumin/phosphate-buffered saline for 90 min on a shaker at 37 °C and incubation with streptavidin-horseradish peroxidase (1:200, Sigma) in 1% bovine serum albumin/phosphate-buffered saline for 30 min with shaking. Diaminobenzidine (DAB) solution (Sigma) was then added and incubated for 10 min at 20 °C. The cells were counterstained using haematoxylin and mounted with Vectamount Permanent Mounting medium (Vector Laboratories). Ten fields of view per slide were imaged using a Leica DM LB2 upright fluorescence microscope.

### Flow cytometry

For flow cytometry cells were resuspended in phosphate-buffered saline and stained for 15 min with CD44-PE (BD Biosciences G44-26) and CD133-APC (Miltenyi Biotec, Bisley, UK, AC133) antibodies. Samples were analysed using a FACS Analyser Cyan ADP (Beckman Coulter, Brea, CA, USA). Dead cells were gated out and at least 10,000 live events were analysed using Summit software v4 (Beckman Coulter).

## References

[bib1] Soufi A, Jayaraman PS. PRH/Hex: an oligomeric transcription factor and multifunctional regulator of cell fate. Biochem J 2008; 412: 399–413. 18498250 10.1042/BJ20080035 PMC2570084

[bib2] Noy P, Williams H, Sawasdichai A, Gaston K, Jayaraman PS. PRH/Hhex controls cell survival through coordinate transcriptional regulation of vascular endothelial growth factor signaling. Mol Cell Biol 2010; 30: 2120–2134. 20176809 10.1128/MCB.01511-09 PMC2863580

[bib3] Kershaw R, Siddiqui Y, Roberts D, Jayaraman PS, Gaston K. PRH/Hhex inhibits the migration of breast and prostate epithelial cells through direct transcriptional regulation of Endoglin. Oncogene 2014; 33: 5592–5600. 24240683 10.1038/onc.2013.496

[bib4] Williams H, Jayaraman PS, Gaston K. DNA wrapping and distortion by an oligomeric homeodomain protein. J Mol Biol 2008; 383: 10–23. 18755198 10.1016/j.jmb.2008.08.004

[bib5] Brickman JM, Jones CM, Clements M, Smith JC, Beddington RS. Hex is a transcriptional repressor that contributes to anterior identity and suppresses Spemann organiser function. Development 2000; 127: 2303–2315. 10804173 10.1242/dev.127.11.2303

[bib6] Marfil V, Blazquez M, Serrano F, Castell JV, Bort R. Growth-promoting and tumourigenic activity of c-Myc is suppressed by Hhex. Oncogene 2014; 34: 3011–3022. 25220416 10.1038/onc.2014.240

[bib7] Marfil V, Moya M, Pierreux CE, Castell JV, Lemaigre FP, Real FX et al. Interaction between Hhex and SOX13 modulates Wnt/TCF activity. J Biol Chem 2010; 285: 5726–5737. 20028982 10.1074/jbc.M109.046649 PMC2820800

[bib8] Topisirovic I, Culjkovic B, Cohen N, Perez JM, Skrabanek L, Borden KL. The proline-rich homeodomain protein, PRH, is a tissue-specific inhibitor of eIF4E-dependent cyclin D1 mRNA transport and growth. EMBO J 2003; 22: 689–703. 12554669 10.1093/emboj/cdg069 PMC140753

[bib9] Jankovic D, Gorello P, Liu T, Ehret S, La SR, Desjobert C et al. Leukemogenic mechanisms and targets of a NUP98/HHEX fusion in acute myeloid leukemia. Blood 2008; 111: 5672–5682. 18388181 10.1182/blood-2007-09-108175

[bib10] D'Elia AV, Tell G, Russo D, Arturi F, Puglisi F, Manfioletti G et al. Expression and localization of the homeodomain-containing protein HEX in human thyroid tumors. J Clin Endocrinol Metab 2002; 87: 1376–1383. 11889211 10.1210/jcem.87.3.8344

[bib11] Puppin C, Puglisi F, Pellizzari L, Manfioletti G, Pestrin M, Pandolfi M et al. HEX expression and localization in normal mammary gland and breast carcinoma. BMC Cancer 2006; 6: 192. 16854221 10.1186/1471-2407-6-192 PMC1550255

[bib12] Soufi A, Noy P, Buckle M, Sawasdichai A, Gaston K, Jayaraman PS. CK2 phosphorylation of the PRH/Hex homeodomain functions as a reversible switch for DNA binding. Nucleic Acids Res 2009; 37: 3288–3300. 19324893 10.1093/nar/gkp197 PMC2691835

[bib13] Noy P, Sawasdichai A, Jayaraman PS, Gaston K. Protein kinase CK2 inactivates PRH/Hhex using multiple mechanisms to de-repress VEGF-signalling genes and promote cell survival. Nucleic Acids Res 2012; 40: 9008–9020. 22844093 10.1093/nar/gks687 PMC3467080

[bib14] Noy P, Gaston K, Jayaraman PS. Dasatinib inhibits leukaemic cell survival by decreasing PRH/Hhex phosphorylation resulting in increased repression of VEGF signalling genes. Leukemia Res 2012; 36: 1434–1437. 22874537 10.1016/j.leukres.2012.07.013 PMC3462996

[bib15] Litchfield DW. Protein kinase CK2: structure, regulation and role in cellular decisions of life and death. Biochem J 2003; 369: 1–15. 12396231 10.1042/BJ20021469 PMC1223072

[bib16] Yenice S, Davis AT, Goueli SA, Akdas A, Limas C, Ahmed K. Nuclear casein kinase 2 (CK-2) activity in human normal, benign hyperplastic, and cancerous prostate. Prostate 1994; 24: 11–16. 7507238 10.1002/pros.2990240105

[bib17] Lang SH, Sharrard RM, Stark M, Villette JM, Maitland NJ. Prostate epithelial cell lines form spheroids with evidence of glandular differentiation in three-dimensional Matrigel cultures. Br J Cancer 2001; 85: 590–599. 11506501 10.1054/bjoc.2001.1967 PMC2364090

[bib18] Berthon P, Cussenot O, Hopwood L, Leduc A, Maitland N. Functional expression of sv40 in normal human prostatic epithelial and fibroblastic cells - differentiation pattern of nontumorigenic cell-lines. Int J Oncol 1995; 6: 333–343. 21556542 10.3892/ijo.6.2.333

[bib19] Trembley JH, Wang G, Unger G, Slaton J, Ahmed K. Protein kinase CK2 in health and disease: CK2: a key player in cancer biology. Cell Mol Life Sci 2009; 66: 1858–1867. 19387548 10.1007/s00018-009-9154-y PMC4385580

[bib20] Collins AT, Berry PA, Hyde C, Stower MJ, Maitland NJ. Prospective identification of tumorigenic prostate cancer stem cells. Cancer Res 2005; 65: 10946–10951. 16322242 10.1158/0008-5472.CAN-05-2018

[bib21] Richardson GD, Robson CN, Lang SH, Neal DE, Maitland NJ, Collins AT. CD133, a novel marker for human prostatic epithelial stem cells. J Cell Sci 2004; 117: 3539–3545. 15226377 10.1242/jcs.01222

[bib22] Deep G, Jain AK, Ramteke A, Ting H, Vijendra KC, Gangar SC et al. SNAI1 is critical for the aggressiveness of prostate cancer cells with low E-cadherin. Mol Cancer 2014; 13: 37. 24565133 10.1186/1476-4598-13-37 PMC3937432

[bib23] Li P, Yang R, Gao WQ. Contributions of epithelial-mesenchymal transition and cancer stem cells to the development of castration resistance of prostate cancer. Mol Cancer 2014; 13: 55. 24618337 10.1186/1476-4598-13-55 PMC3975176

[bib24] Desjobert C, Noy P, Swingler T, Williams H, Gaston K, Jayaraman PS. The PRH/Hex repressor protein causes nuclear retention of Groucho/TLE co-repressors. Biochem J 2009; 417: 121–132. 18713067 10.1042/BJ20080872 PMC2605961

[bib25] Swingler TE, Bess KL, Yao J, Stifani S, Jayaraman PS. The proline-rich homeodomain protein recruits members of the Groucho/Transducin-like enhancer of split protein family to co-repress transcription in hematopoietic cells. J Biol Chem 2004; 279: 34938–34947. 15187083 10.1074/jbc.M404488200

[bib26] Soufi A, Smith C, Clarke AR, Gaston K, Jayaraman PS. Oligomerisation of the developmental regulator proline rich homeodomain (PRH/Hex) is mediated by a novel proline-rich dimerisation domain. J Mol Biol 2006; 358: 943–962. 16540119 10.1016/j.jmb.2006.02.020

