# Supplementary material for: CK2 abrogates the inhibitory effects of PRH/HHEX on prostate cancer cell migration and invasion and acts through PRH to control cell proliferation
Source: Oncogenesis. 2017 Jan 30;6(1):e293–. doi: 10.1038/oncsis.2016.82 (PMC5294245; doi:10.1038/oncsis.2016.82)
Supplement: Supplementary Information [file oncsis201682x1.pdf]

Figure S1

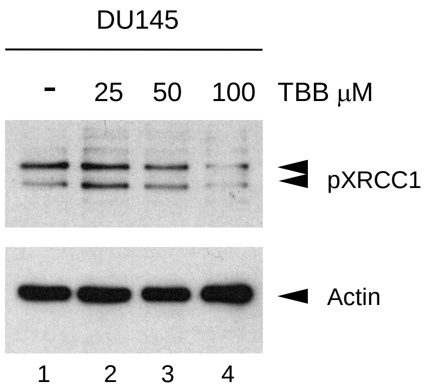

Figure S1 TBB inhibits CK2 in DU145 cells.
DU145 cells were treated with either 10% DMSO (1) or increasing concentrations of TBB in DMSO. Western blotting was then used to examine the levels of pXRCC1. Actin was used as a loading control. Representative of three independent experiments.

Figure S2

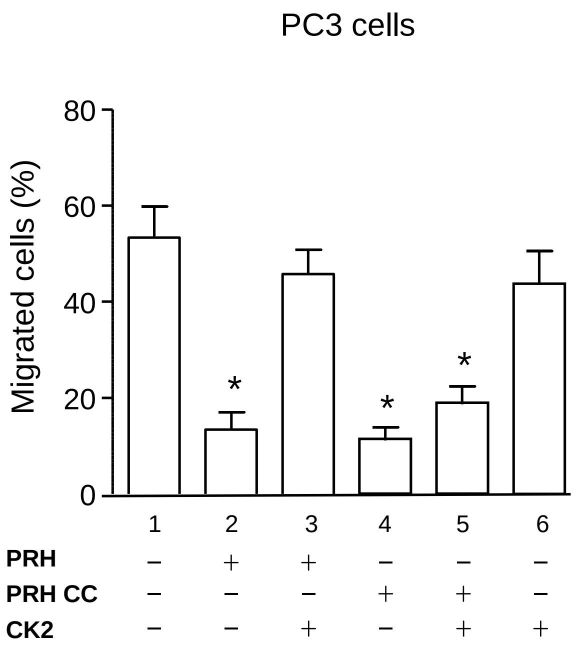

Figure S2 CK2 abrogates the effects of PRH on the migration of PC3 cells. PC3 cells were transiently transfected with a GFP expression vector alone (1) or cotransfected with vectors expressing GFP, PRH or PRH CC and CK2 subunits (2-6). Twenty four hrs post-transfection the cells were plated in Transwell chambers with a 2%:10% serum gradient. After 12 hrs the green cells on the top and bottom surfaces of the filter were counted using microscopy. Cells in ten fields were counted in three independent experiments to determine the percentage of migrated cells. *$p<0.01$. One-way ANOVA.
